# Anticipated Negative Responses by Students to Possible Ebola Virus Outbreak, Guangzhou, China

**DOI:** 10.3201/eid2201.150898

**Published:** 2016-01

**Authors:** Joseph T.F. Lau, Zixin Wang, Yoona Kim, Jing Gu, Anise M.S. Wu, Qianling Zhou, Chun Hao, Perry Cheng, Yuantao Hao

**Affiliations:** Centre for Medical Anthropology and Behavioral Health, Sun Yat-sen University, Guangzhou, China (J.T.F. Lau);; Shenzhen Research Institute, The Chinese University of Hong Kong, Shenzhen, China (J.T.F. Lau);; Jockey Club School of Public Health and Primary Care, The Chinese University of Hong Kong, Hong Kong, China (J.T.F. Lau, Z. Wang, Y. Kim, Q. Zhou, P. Cheng);; Sun Yat-sen University School of Public Health, Guangzhou, China (J. Gu, C. Hao, Y. Hao);; University of Macau, Macau, China (A.M.S. Wu)

**Keywords:** Ebola virus, Ebola virus disease, EVD, China, perceptions, community responses, preparedness, emotions, avoidance, misconceptions, viruses

**To the Editor:** In 2014, a serious Ebola virus disease (EVD) outbreak occurred in West Africa (*1*). In a study on EVD-related perceptions, 85% of US respondents mistakenly believed that EVD could be transmitted through airborne droplets from patients’ sneezes or coughs (*2*). EVD-related panic was reported in the United States (*3*) and the United Kingdom (*4*). 

During November 15–December 20, 2014, we conducted a cross-sectional survey of 1,295 undergraduate students in Guangzhou, China, where the population of immigrants from Africa is high, who had heard of EVD (Technical Appendix). Our aim was to measure students’ anticipated negative emotional responses and avoidance activities (dependent variables) to a possible outbreak of EVD (*5*). We constructed scales for the dependent and independent variables to assess EVD-related perceptions: 1) misconceptions/knowledge about transmission modes, 2) scenarios of an EVD outbreak in Guangzhou (chances, severity, control), 3) efficacy of preventive measures and self-protection, and 4) public stigma toward EVD survivors. MLwiN 2.30 (Centre for Multilevel Modeling, University of Bristol, Bristol, UK) was used for multilevel regression analyses (Technical Appendix).

We analyzed data from 1,155 (89.2%) students who have heard of EVD. To the example of 2–3 EVD cases detected in Guangzhou, 31.0% showed >4 types of anticipated negative emotions (e.g., fear, panic, worry); 59.5% showed >3 types of anticipated unnecessary avoidance. Most (80.0%) indicated >1 misconception regarding transmission mode (e.g., believed it was droplet or waterborne) but knew that direct contact with the corpse of an infected person (69.0%) and body fluids (81.4%) could lead to infection and perceived EVD as fatal (85.6%,) and highly infectious (81.6%). About half of respondents believed that effective treatment and a vaccine were unavailable (51.9% and 59.1%, respectively); 22.2% anticipated EVD outbreaks among Africans in Guangzhou (during the next 12 months). Many students perceived severe consequences if a small EVD outbreak occurred in Guangzhou and believed an outbreak would have a high fatality rate (70.5%), EVD is highly infectious (65.4%), an outbreak would be of long duration (47.5%), and the number of infected persons would be high (39.9%); 52.5%–79.2% of respondents lacked confidence in the government’s ability to control an outbreak (e.g., ability to provide adequate vaccines, medication, protective gear). Half or more of respondents believed that restricting travel by Africans to and from Africa and avoiding visiting African-inhabited areas were effective means of prevention. About 40% were confident that they could protect themselves or family members from EVD (Technical Appendix Tables 1, 2).

Older age, female sex, longer school years, and rural origin were associated with negative emotional responses, avoidance, or both (Technical Appendix Table 3). In multivariate analyses that adjusted for significant background variables, we found positive associations between both dependent variables and the following independent variables: perceived fatality of EVD, perceived nonavailability of treatment, misconceptions regarding modes of transmission, perceived severity of a Guangzhou outbreak, perceived efficacy of restricting Africans’ travel, perceived efficacy of avoiding African-inhabited areas, and public stigma toward EVD survivors. Confidence in governmental control was negatively associated with both dependent variables. Some variables were positively associated with emotional response but not avoidance (perceived irreversible harm, perceived chance of outbreak in Guangzhou and in other parts in China, perceived self-efficacy for protection); 2 variables (perceived nonavailability of vaccine and knowledge of transmission mode) were positively associated with avoidance measures but not with emotional responses (Table).

**Table:** Factors associated with anticipated responses to EVD, adjusted for sociodemographic variables, Guangzhou, China, 2014*

Factor	Emotional Response to Ebola Scale†			Unnecessary Avoidance Scale‡	
β (SE)	p value		β (SE)	p value
Perceived severity of EVD					
EVD is fatal	1.270 (0.928)	0.171		0.855 (0.388)	**0.027**
EVD causes irreversible harm to physical health	2.647 (0.637)	**<0.001**		0.504 (0.064)	0.064
Perceived fatality of EVD	2.545 (0.635)	**<0.001**		1.177 (0.269)	**<0.001**
Perceived high infectivity of EVD	1.568 (0.842)	0.063		1.273 (0.350)	**<0.001**
Treatment and vaccine availability					
Treatment not available	2.143 (0.639)	**<0.001**		1.108 (0.271)	**<0.001**
Vaccine not available	1.236 (0.654)	0.059		0.786 (0.276)	**0.005**
Misconceptions and knowledge about modes of transmission of EVD					
Misconceptions about Mode of Transmission Scale	0.406 (0.113)	**<0.001**		0.214 (0.048)	**<0.001**
Knowledge about Mode of Transmission Scale	0.285 (0.171)	0.095		0.369 (0.071)	**<0.001**
Perceptions related to EVD outbreak					
Chances of Outbreak Scale—Guangzhou	0.688 (0.091)	**<0.001**		0.064 (0.039)	0.100
Perceived Chances of Outbreak Scale—Other Parts of China	0.986 (0.189)	**<0.001**		0.151 (0.081)	0.062
Perceived Severity of Outbreak in Guangzhou Scale	0.825 (0.072)	**<0.001**		0.222 (0.031)	**<0.001**
Confidence in Governmental Control Scale	−1.024 (0.086)	**<0.001**		−0.192 (0.038)	**<0.001**
Perceived efficacy and self-efficacy					
Perceived Efficacy of Restricting Africans Travel Scale	1.003 (0.176)	**<0.001**		0.543 (0.073)	**<0.001**
Perceived Efficacy of Avoidance Scale	0.544 (0.138)	**<0.001**		0.595 (0.056)	**<0.001**
Perceived Self-Efficacy for Protection against EVD Scale	−0.571 (0.145)	**<0.001**		−0.112 (0.062)	0.070
Public stigma toward EVD survivors					
Public Stigma Scale	0.231 (0.032)	**<0.001**		0.125 (0.013)	**<0.001**

*Among participants who had heard of EVD (n = 1,155). Bold indicates significance. β, multilevel linear regression coefficient adjusted by significant background variables; EVD, Ebola virus disease. †Anticipated Emotional Response to Ebola Scale items included the following: “If there are 2–3 EVD cases in Guangzhou, how likely would you 1) worry about getting infected with EVD, 2) worry about family members getting infected with EVD, 3) be scared, 4) be uneasy, 5) be in panic, 6) feel helpless, 7) be depressed, 8) have insomnia, 9) be distressed, 10) have fluctuating emotions, and 11) be emotionally disturbed.” Response categories: 1 = very unlikely, 5 = very likely. ‡Unnecessary Avoidance Scale items included the following: “If there are 2–3 EVD cases in Guangzhou, how likely would you be to 1) avoid going to other cities, 2) avoid going to work, 3) avoid going out if unnecessary, 4) avoid going to crowded places, 5) avoid going to hospitals, and 6) avoid taking airplanes.” Response categories: 1 = very unlikely, 5 = very likely.

Because EVD causes serious physical harm, negative emotional responses and unnecessary avoidance practices were anticipated. Such negative community responses might cause individual and societal harm, as witnessed during the epidemic of severe acute respiratory syndrome (*6*). Misconceptions concerning transmission modes were prevalent and significantly correlated with both dependent variables. More than 80% of respondents perceived that the virus was highly infectious, another significant factor. 

About 20% of participants believed that an EVD outbreak would occur in Guangzhou in the next year. Among all participants, many anticipated severe outcomes but were not confident that the government was prepared for and could control such an outbreak. 

The concentration of immigrants from Africa in this region might have increased perceived chances of an EVD outbreak and thus lead to avoidance of this population. The high percentages of those who believed that restricting Africans’ travel was effective also might result in discrimination. 

Public stigmatization toward EVD survivors, another significant factor, was a prominent attitude (*7*,*8*). Fear, misconceptions, and perceived likelihood of EVD to cause death may lead to patient stigmatizing, which could hinder case detection and patients’ service seeking. The relationship between stigmatization and EVD-related perceptions should be investigated. 

The study’s limitations included the inability to assess real responses, inability to generalize findings to all university students and the general public, and the use of scales that had not been validated. Also, some students might have given exaggerated responses.

In summary, misconceptions and perceptions regarding EVD may result in negative community responses in Guangzhou. Health education is needed to clarify that EVD is not airborne or waterborne or highly infectious and that avoidance is not an effective preventive measure. In addition, the government should start developing and publicizing its preparedness plans.

**Technical Appendix.** Detailed methods and background variables of the study participants in Guangzhou, China, the frequency distribution of items related to Ebola virus disease (EVD), associations between sociodemographic factors and anticipated responses to an EVD outbreak, and univariate associations between independent variables and anticipated responses to EVD by participants.
