# Supplementary material for: Anticipated Negative Responses by Students to Possible Ebola Virus Outbreak, Guangzhou, China
Source: Emerg Infect Dis. 2016 Jan;22(1):154–6. doi: 10.3201/eid2201.150898 (PMC4696696; doi:10.3201/eid2201.150898)
Supplement: Supplementary file 1 — Technical Appendix. Detailed methods and background variables of the study participants in Guangzhou, China, the frequency distribution of items related to Ebola virus disease (EVD), associations between sociodemographic factors and anticipated responses to an EVD outbreak, and univariate associations between independent variables and anticipated responses to EVD by participants. [file 15-0898-Techapp-s1.pdf]

Article DOI: http://dx.doi.org/10.3201/eid2201.150898

Anticipated Negative Responses by University Students to Possible Ebola Outbreak, Guangzhou, China

Technical Appendix

Methods

Sampling and Data Collection

This cross-sectional study was conducted during November 15–December 20, 2014, in 2 major universities in Guangzhou that had 41,000 and 50,000 students. Four undergraduate core classes were randomly selected from all related classes of each of 6 schools (public health, clinical medicine, chemical industry, mathematics and computer, sociology, politics and public affairs management) of the 2 universities. All students attending the selected classes were invited to self-administer an anonymous questionnaire in classrooms. They were reminded not to fill out the questionnaire twice. Research assistants read a statement indicating that participation is voluntary, refusal would have no effect on them, and data would only be used for research purposes. No names were entered in the questionnaire; written informed consent was recorded separately. No incentive was involved. Ethics approval was obtained from the ethics committee of The Chinese University of Hong Kong. Of the 1,888 students (30% of all 7 schools' students) invited to join the study, 1,295 (68.6%; range 45.5%–78.9% in the 7 schools) completed the questionnaire (refusal: 479 [25.3%]; incomplete: 114 [6.0%]).

Measures

Dependent Variables

The first dependent variable was the 11-item Anticipated Emotional Response Scale (AERES), constructed to assess anticipated emotional responses if 2–3 Ebola virus disease (EVD) cases were detected in Guangzhou. A sample item is the following: "If there are 2–3 EVD cases in Guangzhou, how likely would you be to panic?" (Cronbach $\alpha = 0.953$). The

second dependent variable was the 6-item Unnecessary Avoidance Scale (UAS) (Cronbach α = 0.775). Ratings were made on Likert scales (1 = very unlikely to 5 = very likely).

Independent Variables

Four items were used to assess the perceived severity of EVD; 3 were rated on 3-point Likert scales and 1 asked about perceived fatality rate of EVD. Questions were also asked regarding the perceived availability of effective treatment and vaccine for EVD. The 6-item Misconceptions about Mode of Transmission Scale (MISTS) and the 4-item Knowledge on Modes of Transmission Scale (KTS) were constructed for this study.

Three scales were constructed to assess perceptions on anticipated scenarios of a potential EVD outbreak in Guangzhou, including the following: 1) the 4-item Perceived Chance of Outbreak in Guangzhou Scale (PCOS_GZ), 2); the 6-item Perceived Severity of Outbreak in Guangzhou Scale (PSO_GZ); and 3) the 5-item Confidence in Governmental Control Scale (CGCS). Another 2-item scale, the Perceived Chance of Outbreak in Other Parts of China Scale (PCOS_OC) was constructed to assess perceived chance of outbreak in other parts of China. Response categories of these scales ranged from 1 (very low or strongly disagree) to 5 (very high or strongly agree).

The 2-item Perceived Efficacy of Restricting Africans' Travel Scale (PERAT) and the 4-item Perceived Efficacy of Avoidance Scale (PEAS) rated perceived efficacy of such measures; response categories ranged from 1 (very ineffective) to 5 (very effective). The Perceived Self-efficacy for Protection against EVD Scale (PSEP) had 2 items, with responses ranging from 1 (not confident at all) to 5 (totally confident). The Public Stigma Scale, which has been used to assess stigma towards schizophrenia (*1*) and mental illness (*2*) in some Chinese populations, was modified and used in this study.

Exploratory factor analysis found single factors for all of the constructed scales, explaining 46.8% and 82.1% of the total variances. Cronbach α ranged from 0.642 to 0.953. (Details and items are shown in Technical Appendix Table 2.)

Statistical Analysis

Descriptive characteristics of the sample were analyzed by using SPSS 16.0 (IBM Corp., Armonk, NY, USA). A multilevel regression model was used to examine factors associated with

the 2 dependent variables among the students. Individual students were selected by a stratified cluster sampling method at the class level. The random intercepts model was therefore used, in which intercepts of the regression model were allowed to vary across classes. Such a model can account for intracorrelated nested data. The 2-level linear regression model (level 1: classes; level 2: students) was performed by using MLwiN 2.30 (Centre for Multilevel Modeling, University of Bristol, Bristol, UK). First, univariate associations between independent variables and dependent variables (AERES and UAS) were tested. After adjusting for significant sociodemographic variables, regression coefficients (β) were obtained; p values <0.05 were considered statistically significant.

References

1. Mak WWS, Leung SYC. Common sense model on public stigma: implications on social distance and help-seeking among Chinese community adults. Hong Kong: The Chinese University of Hong Kong; 2008.

2. Chan JY, Mak WW, Law LS. Combining education and video-based contact to reduce stigma of mental illness: “The Same or Not the Same” anti-stigma program for secondary schools in Hong Kong. Soc Sci Med. 2009;68:1521–6. PubMed http://dx.doi.org/10.1016/j.socscimed.2009.02.016

Technical Appendix Table 1. Background characteristics of study participants, Guangzhou, China, 2014*

Characteristic	No. (%) all participants, N = 1,295	No. (%) participants who had heard of EVD, n = 1,155	No. (%) participants who had not heard of EVD, n = 140	p value†
Age, y, mean ± SD	19.94 ± 1.55	19.90 ± 1.52	20.26 ± 1.77	**0.009**
Sex				0.057
M	698 (54.3)	613 (53.4)	85 (62.0)	
F	587 (45.7)	535 (46.6)	52 (38.0)	
School affiliations				**<0.001**
Public Health	123 (9.5)	119 (10.3)	4 (2.9)	
Clinical Medicine	314 (24.2)	295 (25.5)	19 (13.6)	
Chemical Industry	475 (36.7)	424 (36.7)	51 (36.4)	
Sociology	97 (7.5)	91 (7.9)	6 (4.3)	
Politics and Public Affairs Management	84 (6.5)	46 (4.0)	38 (27.1)	
Mathematics and Computer	202 (15.6)	180 (15.6)	22 (15.7)	
School year				0.099
1	308 (23.8)	275 (23.8)	33 (23.6)	
2	400 (30.9)	345 (29.9)	55 (39.3)	
3	288 (22.3)	260 (22.5)	28 (20.0)	
4	295 (22.8)	274 (23.7)	24 (17.1)	
Place of origin				0.454
Guangzhou	139 (10.6)	121 (10.5)	18 (12.9)	
Other places in Guangdong	531 (41.1)	470 (40.8)	61 (43.6)	
Outside Guangdong	623 (48.2)	562 (48.7)	61 (43.6)	
Rural/urban origin				0.766
Large city	379 (29.3)	341 (29.6)	38 (27.3)	
Medium-sized or small city	434 (33.6)	385 (33.4)	49 (35.3)	
Town	230 (17.8)	202 (17.5)	28 (20.1)	
Village	249 (19.3)	225 (19.5)	24 (17.3)	

Characteristic	No. (%) all participants, N = 1,295	No. (%) participants who had heard of EVD, n = 1,155	No. (%) participants who had not heard of EVD, n = 140	p value†
Length of stay in Guangzhou, y, mean ± SD	3.51 ± 5.31	3.51 ± 5.28	3.46 ± 5.56	0.905

*EVD, Ebola virus disease; n = 1,155.
†Independent sample *t* test for continuous variables and χ2 test for categorical variables.

Technical Appendix Table 2. Frequency distribution of items related to EVD among participants who had heard of EVD, Guangzhou, China, 2014*†

Response	No. patients or mean score	% Patients or SD
Anticipated emotional response if 2–3 EVD cases in Guangzhou (% Likely/Very likely)		
Worry about getting infected with EVD	648	56.1
Worry about family members getting infected with EVD	535	46.3
Scared	388	33.6
Uneasy	389	33.7
Panic	326	28.2
Helpless	252	21.8
Depressed	220	19.0
Insomnia	131	11.3
Distressed	194	16.8
Emotional fluctuation	162	14.0
Emotional disturbance	160	13.9
Scale score		
Anticipated Emotional Response Scale (AERES, 11 items)†	27.9	11.1
Unnecessary avoidance if 2–3 EVD cases in Guangzhou (% Likely/Very likely)		
Avoid going to other cities	792	68.6
Avoid going to work	155	13.4
Avoid going out unless necessary	609	52.7
Avoid going to crowded places	853	73.9
Avoid going to hospitals	562	48.7
Avoid taking airplanes	381	33.0
Scale score		
Unnecessary Avoidance Scale (UAS, 6 items)‡	19.1	4.7
Perceived severity of EVD		
EVD is fatal		
Disagree/uncertain	166	14.4
Agree	989	85.6
EVD causes irreversible harm of physical health		
Disagree/uncertain	594	51.4
Agree	561	48.6
Fatality rate of EVD		
< 70%/uncertain	593	51.3
≥70%	562	48.7
EVD has high infectivity		
Disagree/Uncertain	212	18.4
Agree	943	81.6
Availability of treatment and vaccines		
Effective treatment not available		
Disagree/uncertain	556	48.1
Agree	599	51.9
Effective vaccine not available		
Disagree/Uncertain	472	40.9
Agree	683	59.1
Misconceptions and knowledge about modes of transmission of EVD		
Misconceptions		
Whether EVD can spread by the following routes? (% agree)		
Airborne	213	18.4
Droplets	725	62.8
Mosquitoborne	394	34.1
Direct contact with bird	317	27.4
Foodborne	343	29.7
Waterborne	533	46.1
Scale score		
Misconceptions about Mode of Transmission Scale (MISTS, 6 items)§	12.3	2.8
Knowledge		
Whether EVD can spread by the following routes? (% Agree)		
Direct contact with infected people	557	48.2
Direct contact with body fluid of infected persons	940	81.4

Response	No. patients or mean score	% Patients or SD
Direct contact with body of animal that died of Ebola	659	57.1
Direct contact with body of deceased infected persons	797	69.0
Scale score		
Knowledge about Mode of Transmission Scale (KTS, 4 items)¶	10.1	1.9
Perceptions related to EVD outbreak		
Perceived chances of EVD outbreak in Guangzhou in next year (PCOS_GZ) (% High/Very high)		
Perceived chance of EVD outbreak among Africans living in GZ	256	22.2
Perceived chance of EVD outbreak among Chinese living in GZ	177	15.3
Perceived chance of large scale EVD outbreak in GZ	90	7.8
Perceived chance of EVD outbreak among healthcare workers in GZ	188	16.3
Scale score		
Perceived Chances of Outbreak Scale–Guangzhou (PCOS_GZ, 4 items)#	10.0	3.5
Perceived chances of EVD outbreak in other places in China in next year (% High/Very high)		
Perceived chance of EVD outbreak among Africans living in other places in China	129	11.2
Perceived chance of EVD outbreak among Chinese living in other places in China	106	9.2
Scale score		
Perceived Chances of Outbreak Scale–Other Places in China (PCOS_OC, 2 items)**	4.9	1.7
Perceived severity of EVD outbreak in Guangzhou		
Perceived consequences of EVD outbreak in Guangzhou (% Agree/Strongly agree)		
High mortality rate of infected persons	814	70.5
Long duration of the outbreak	549	47.5
... Highly infectious	755	65.4
Huge number of infected persons	461	39.9
Ineffectiveness of treatment	453	39.2
Ineffectiveness of prevention measures	312	27.0
Scale score		
Perceived Severity of Outbreak in Guangzhou Scale (PSO_GZ, 6 items)††	20.0	4.3
Confidence in governmental control of EVD outbreak (% Agree/Strongly agree)		
Guangzhou government would be able to control outbreak	515	44.6
Guangzhou government would have vaccines to control outbreak	240	20.8
Guangzhou government would have enough medication to control outbreak	473	40.9
Healthcare workers in Guangzhou would have enough protective equipment for themselves	549	47.5
Hospitals in Guangzhou would have enough quarantine measures to control outbreak	602	52.2
Scale score		
Confidence in Governmental Control Scale (CGCS, 5 items)‡‡	16.1	3.6
Perceived efficacy and self-efficacy		
Perceived efficacy of restricting Africans' travel in preventing EVD(% Effective/Very effective)		
Restricting Africans coming to Guangzhou	549	47.5
Restricting Africans living in Guangzhou coming back and forth to Africa	695	60.2
Scale score		
Perceived Efficacy of Restricting Africans' Travel Scale (PERAT, 2 items)§§	6.9	1.8
Perceived efficacy of avoidance in preventing EVD (% Effective/Very effective)		
Avoid going to African-inhabited areas in Guangzhou	689	59.7
Avoid going to countries having an EVD outbreak	964	83.5
Avoid going to crowded places	834	72.2
Avoid taking airplanes	193	16.7
Scale score		
Perceived Efficacy of Avoidance Scale (PEAS, 4 items)¶¶	13.9	2.4
Perceived self-efficacy for protection against EVD (% Confident/Very confident)		
Confident in protecting oneself from EVD	403	34.9
Confident in protecting family members from EVD	441	38.2
Scale score		
Perceived Self-Efficacy for Protection against EVD Scale (PSEP, 2 items)##	6.3	2.2
Public stigma toward EVD survivors		
Public Stigma Scale (20 items)***	64.5	10.2

*EVD, Ebola virus disease; n = 1,155.
†Cronbach α = 0.953, 1 factor was identified by exploratory factor analysis (EFA), which explained 82.1% of total variance.
‡Cronbach α = 0.775, 1 factor was identified by EFA, which explained 46.8% of total variance.
§Cronbach α = 0.650, 1 factor was identified by EFA, which explained 55.8% of total variance.
¶Cronbach α = 0.642, 1 factor was identified by EFA, which explained 53.8% of total variance.
#Cronbach α = 0.884, 1 factor was identified by EFA, which explained 74.2% of total variance.
**Cronbach α = 0.822.
††Cronbach α = 0.807, 1 factor was identified by EFA, which explained 70.8% of total variance.
‡‡Cronbach α = 0.793, 1 factor was identified by EFA, explained 57.3% of total variance.
§§Cronbach α = 0.811.
¶¶Cronbach α = 0.769, 1 factor was identified by EFA, which explained 47.2% of total variance.
##Cronbach α = 0.885.
***Cronbach α = 0.749, 1 factor was identified by EFA, which explained 67.2% of total variance.

Technical Appendix Table 3. Associations between sociodemographic factors and anticipated responses to EBV outbreak among participants who had heard of EVD, Guangzhou, China, 2014*

	AERES		UAS	
Factor	β (SE)	p	β (SE)	p
Age, y	0.588 (0.236)	**0.013**	0.125 (0.108)	0.246
Sex	2.543 (0.662)	**<0.001**	0.740 (0.286)	**0.010**
School affiliations	–0.254 (0.260)	0.327	–0.159 (0.121)	0.190
School year	1.187 (0.338)	**<0.001**	0.591 (0.145)	**<0.001**
Place of origin	–1.244 (1.050)	0.238	–0.045 (0.445)	0.920
Rural/urban origin	0.198 (0.298)	0.509	0.291 (0.127)	**0.022**
Length of stay in Guangzhou, y	–0.058 (0.061)	0.342	0.000 (0.026)	1.000

*n = 1,155.

*EVD, Ebola virus disease; AERES, Anticipated Emotional Response to Ebola Scale; UAS, Unnecessary Avoidance Scale; β, multilevel univariate linear regression coefficient; bold, p<0.05.

Technical Appendix Table 4. Univariate associations between independent variables and anticipated responses to EVD, among participants who had heard of EVD, Guangzhou, China, 2014*†

	AERES		UAS	
Variable	β (SE)	p	β (SE)	p
Perceived severity of EVD				
Fatal	1.183 (0.924)	0.200	0.793 (0.393)	**0.044**
Causes irreversible harm to physical health	2.689 (0.638)	**<0.001**	0.502 (0.272)	0.064
Perceived fatality of EVD	2.570 (0.639)	**<0.001**	1.176 (0.270)	**<0.001**
Perceived high infectivity of EVD	1.869 (0.833)	**0.025**	1.393 (0.352)	**<0.001**
Treatment and vaccine				
Nonavailability of treatment	2.403 (0.642)	**<0.001**	1.162 (0.272)	**<0.001**
Nonavailability of vaccine	1.461 (0.654)	**0.026**	0.813 (0.277)	**0.003**
Misconceptions and knowledge about modes of transmission of EVD				
Misconceptions about Mode of Transmission Scale (MISTS)	0.390 (0.113)	**<0.001**	0.207 (0.048)	**<0.001**
Knowledge about Mode of Transmission Scale (KTS)	0.321 (0.171)	0.061	0.385 (0.072)	**<0.001**
Perceptions related to EVD outbreak				
Perceived Chances of Outbreak Scale-Guangzhou (PCOS-GZ)	0.739 (0.090)	**<0.001**	0.076 (0.039)	0.052
Perceived Chances of Outbreak Scale-Other parts in China (PCOS-OC)	1.084 (0.188)	**<0.001**	0.180 (0.081)	**0.026**
Perceived Severity of Outbreak in Guangzhou Scale (PSO-GZ)	0.846 (0.072)	**<0.001**	0.232 (0.031)	**<0.001**
Confidence in Governmental Control Scale (CGCS)	--1.067 (0.085)	**<0.001**	-0.208 (0.038)	**<0.001**
Perceived efficacy and self-efficacy				
Perceived Efficacy of Restricting Africans' Travel Scale (PERAT)	1.122 (0.174)	**<0.001**	0.568 (0.072)	**<0.001**
Perceived Efficacy of Avoidance Scale (PEAS)	0.619 (0.136)	**<0.001**	0.610 (0.055)	**<0.001**
Perceived Self-Efficacy for Protection against EVD Scale (PSEP)	–0.564 (0.146)	**<0.001**	–0.107 (0.062)	0.085
Public Stigma towards EVD survivors				
Public Stigma Scale	0.234 (0.032)	**<0.001**	0.126 (0.013)	**<0.001**

*n = 1,155.

†EVD, Ebola virus disease; AERES, Anticipated Emotional Response to Ebola Scale; UAS, Unnecessary Avoidance Scale; β, multilevel univariate linear regression coefficient; bold, p<0.05.
